# Association of vitamin D deficiency and premature coronary artery disease

**DOI:** 10.22088/cjim.10.1.80

**Published:** 2019

**Authors:** Hamidreza Norouzi, Naghmeh Ziaie, Mehrdad Saravi, Alireza Norouzi, Somayye Noei-teymoordash, Fazilat Jokar-Darzi, Fateme Norouzi, Maede Rajabi-Fumashi, Farbod Zahedi-Tajrishi, Shahram Norouzi

**Affiliations:** 1 Social Determinants of Health Research Center, Health Research Institute, Babol University of Medical Sciences, Babol, Iran; 2 Department of Cardiology, Babol University of Medical Sciences, Babol, Iran; 3 Department of Cardiology, Shahid Beheshti University of Medical Sciences, Tehran, Iran; 4 Department of Obstetrics and Gynecology, Iran University of Medical Sciences, Tehran, Iran; 5 Department of Internal Medicine, Babol University of Medical Sciences, Babol, Iran; 6 Department of Midwifery, Tehran University of Medical Sciences, Tehran, Iran; 7 Student Research Committee, Faculty of Medicine, Babol University of Medical Sciences, Babol, Iran; 8 Department of Pediatrics, Shahid Beheshti University of Medical Sciences, Tehran, Iran

**Keywords:** 25 Hydroxy vitamin D, Coronary artery disease, Coronary angiography, Vitamin D deficiency

## Abstract

**Background:**

Evidence suggests hypovitaminosis D is associated with increased risk of coronary artery disease (CAD) and its extent and related risk factors. However, some investigations have produced contrary results. Therefore, we aimed to evaluate the association between serum vitamin D levels and the severity of premature coronary artery involvement.

**Methods:**

This randomized prospective, case-control study was conducted in Babol from April 2013 to June 2017. We collected the demographic data and measured serum 25-OH-D levels of 294 patients (age≤50 years) diagnosed with CAD with coronary angiography as case group as well as 438 age and sex-matched controls. CAD severity was assessed using the Gensini score. Statistical analyses were used to assess the associations and p<0.05 was considered as significant.

**Results:**

The mean serum level of 25-OH-D was 13.12±11.13 and 18.28±8.34 in case and control groups, respectively (P=0.036). In the case group, mean serum vitamin D levels were significantly lower among hypertensives (P=0.018), those with a family history of CVD (P=0.016) and those who used aspirin (P=0.036). The mean Gensini score of patients in the case group was 45.02±23.62 and was higher among men (P=0.022). There was a weak significant correlation between the serum vitamin D levels and the Gensini score (P=0.001 & R=-0.543). The mean Gensini score was not significantly different between patients with deficient (47.02±22.78), insufficient (26.0±21.72) and sufficient (39.0±43.84) vitamin D levels (P>0.05).

**Conclusion:**

The results showed that the lower levels of vitamin D is associated with increased risk and extent of coronary artery involvement as well as some of the risk factors of CAD, including male gender, hypertension and positive family history for CVD.

A huge body of literature about vitamin D has mainly focused on its role in calcium homeostasis and the musculoskeletal system. However, evidence on the presence of vitamin D receptors (VDR) in vascular endothelial cells, cardiac smooth muscle cells and myocardial cells has suggested that vitamin D could potentially affect the cardiovascular system as well (1). During the past few years, multiple studies have proposed vitamin D effects on the inflammatory system and atherothrombosis. Evidence further suggests that hypovitaminosis D is associated with endothelial dysfunction and increased risk of cardiovascular disorders and their related risk factors such as dyslipidemia, hypertension and diabetes mellitus (2-4). 

Among cardiovascular disorders, coronary artery disease (CAD) is notoriously dangerous since it is the most common cause of morbidity and mortality worldwide (5). While several studies have linked vitamin D deficiency with CAD (6-9), some investigations have produced contrary results (10). 

As this controversy continues to persist, we aimed to collect further evidence by investigating the association between serum vitamin D levels and the extent and severity of premature coronary artery involvement.

## Methods

**Study population and characteristics:** We conducted this prospective, randomized, case-control study in Babol Ayatollah Rouhani Hospital from April 2013 to June 2017. Consecutive individuals (aged ≤50 years) admitted to the CCU and cardiology ward with suspected CAD were studied. Patients with renal failure (GFR ≤60cc/min), liver disease, history of malignancy during the past 5 years, those treated with Vitimin D supplements, patients with congenital genetically dyslipidemia, anemia and parathyroid diseases were excluded. The demographic data of the participants such as age, sex, education, smoking status, use of antihyperlipidemic, antidiabetic and antihypertensive drugs were recorded upon admission. The study protocol was approved by the institutional review board of Babol University of Medical Sciences. All patients signed an informed written consent before entering the study.

**Coronary angiography and Gensini scaling score: **All patients underwent coronary angiography at the time of admission, performed by a board-certified cardiologist using the standard Judkins technique. Subjects were then categorized into CAD (case) and non-CAD (control) groups according to the result of their coronary angiogram and the controls were matched for age and sex. In the patient group, CAD severity was assessed by Gensini score, which is based on the percentage of luminal narrowing (25%: 1 point; 50%: 2 points; 75%: 4 points; 90%: 8 points; 99%: 16 points, and total occlusion: 32 points). Each coronary lesion score was calculated using the percentage of luminal narrowing multiplied by coefficient of coronary segment: the left main coronary artery (LMCA) x5; the proximal segment of the left anterior descending coronary artery (LAD) x 2.5; the proximal segment of the circumflex artery (CX) x 2.5; the mid-segment of the LAD x 1.5; the distal segment of the LAD, all segments of the right coronary artery (RCA) and the obtuse marginal artery x 1; and other segments x 0.5. The Gensini score was calculated by the summation of individual coronary segment scores in the case group (11).

**Serum vitamin D assessment:** 25-hydroxyvitamin D is the major circulating form of vitamin D which is formed predominantly in the liver (12). To measure serum 25 (OH) vitamin D levels,a blood sample was taken from each participant after angiography. Serum levels of 25OH-vitamin D were measured by the CLIA method by Liaison kits (Italy). The patients were then divided into three groups based on their serum 25-OH-vit D concentration:

Normal: vit. D level ≥30 ng/ml Insufficient: 30 > vit. D level≥20 Deficient: 20> vit. D level

**Statistical analysis:** Collected data were analyzed by SPSS Version 23 statistical software (IBM Corporation, USA) and a p<0.05 was considered significant. Parametric data were analyzed using unpaired T- test and ANOVA for two and more than two groups. For categorical, nonparametric data, Chi-square test was applied. Mann-Whitney test was used to compare the variables that were not normally distributed. Logistic regression analysis was used to describe the relationship between one dependent binary variable and one nominal variable.

## Results

Table 1 represents the baseline characteristics of both case and control groups. A total of 732 individuals, including 294 patients and 438 controls participated in our study (mean age: 46.88±3.45 and 45.94±3.72, respectively; p>0.05). The patient group and their controls did not show any significant difference in terms of sex (p>0.05). The distribution of vitamin D deficiency among men and women showed no significant difference either (p>0.05). In the case group, 4.1% (12 cases) of patients had sufficient levels of vitamin D, 7.8% (23 cases) subjects had insufficient levels and 88.1% (259 cases) inwere vitamin D deficient. In the control group, on the other hand, 19.9% (87 subjects) of patients had sufficient levels of vitamin D, 10.0% (44) patients had insufficient levels and 70.1% (307) patients were vitamin D deficient. With regard to the distribution of serum vitamin D levels, there was a significant difference between case and control groups (P =0.042). The mean serum level of 25 OH-vit D was 13.12±11.13 in the case group and 18.28± 8.34 in the control group (P=0.036).

**Table 1:** Baseline characteristics of patients and matched control subjects

**Characteristic**		**Cases** **(294)**	**Controls** **(438)**	**P value**
Age, mean(SD)		46.88(3.45)	45.94(3.72)	0.612
Sex	Male (%)	153(52)	193(44.1)	0.548
	Female (%)	141(48)	245(55.9)	
BMI(SD)		26.56(2.19)	27.3(4.7)	0.734
Region of residence	Burgess (%)	288(98)	140(32)	≤ 0.001
	Rural (%)	6(2)	298(68)	
Educational state	Primary education (%)	6(2)	184(42)	≤ 0.001
	Secondary education (%)	124(42.2)	184(42)	
	Academic education (%)	164(55.8)	70(16)	
Smoker (%)		72(24.5)	126(28.8)	0.820
Opium addiction (%)		12(4.1)	36(8.2)	0.678
HTN (%)		162(55.1)	243(55.5)	1
DM (%)		102(34.7)	162(37)	0.830
HLP (%)		150(51)	153(35)	0.687
Family History of CVD (%)		192(65.3)	288(65.8)	1
Drug history	ASA (%)	186(63.3)	297(67.8)	0.835
	Clopidogerel (%)	102(34.7)	144(32.9)	1
	Nitroglycerin (%)	102(34.7)	99(22.7)	0.265
	Statin (%)	138(47)	207(47.3)	1
	ACEI/ARB (%)	138(47)	216(49.3)	1
	Beta-blocker (%)	162(55.1)	225(51.4)	0.841
Vitamin D group	Sufficient (%)	12(4.1)	87(19.9)	0.042
	Insufficient (%)	23(7.8)	44(10)	
	Deficient (%)	259(88.1)	307(70.1)	
First presentation	MI (%)	53(18)	0(0)	0.003
	Unstable angina (%)	71(24.1)	167(38.1)	
	Stable angina (%)	170(57.9)	271(61.9)	

In the case group, the mean serum vitamin D levels were significantly lower among hypertensives (P=0.018), those with a family history of CVD (P=0.016) and those who used aspirin (P=0.036) compared with patients with normotensive patients, those with an insignificant family history of CVD and those who did not consume aspirin, respectively. Other risk factors such as smoking, diabetes mellitus and hyperlipidemia were not significantly associated with lower serum vitamin D concentrations (table 2).

**Table 2:** The relationship between mean of vitamin D level and CAD RFs, drugs and other demographic characteristics in patients

**Characteristic**		**Mean of Vitamin D level**	**SD**	**P value**
Sex	Male	15.54	14.99	0.141
	Female	10.88	5.03	
Region of residence	Burgess	13.12	11.13	≤ 0.001
	Rural	28	-	
Educational state	Primary education	16	-	≤ 0.001
	Secondary education	15.86	15.39	
	Academic education	10.96	5.45	
Smoker (Yes/No)		10.67/13.89	5.34/12.37	0.387
Opium addiction (Yes/No)		10.50/13.23	6.36/11.32	0.738
HTN (Yes/No)		9.74/17.09	4.81/14.77	0.018
DM (Yes/No)		9.35/15.06	6.14/12.63	0.086
HLP (Yes/No)		10.92/15.32	11.24/10.80	0.165
Family History of CVD (Yes/No)		10.31/18.11	5.00/16.44	0.016
Drug history	ASA (Yes/No)	10.55/17.32	5.25/16.17	0.036
	Clopidogerel (Yes/No)	14.76/12.27	13.03/10.13	0.459
	Nitroglycerin (Yes/No)	14.12/12.61	13.11/10.15	0.654
	Statin (Yes/No)	12.35/13.78	11.62/10.88	0.656
	ACEI/ ARB (Yes/No)	12.17/13.93	11.64/10.84	0.585
	Beta-blocker (Yes/No)	14.22/11.83	14.19/5.89	0.454
First presentation	MI (%)	3.43	1.12	0.027
	Unstable angina (%)	13.67	11.93	
	Stable angina (%)	12.72	10.71	
Angiographic results	SVD	16.58	14.88	0.239
	2VD	10.83	5.80	
	3VD	14.85	14.35	
	MVD	4	-	

As mentioned earlier, we also divided the participants of our study into deficient, insufficient and sufficient vit.D level groups. The mean Gensini score in these groups was 47.02±22.78, 26.0±21.72 and 39.0±43.84, respectively (P > 0.05). The mean Gensini score was higher among men compared with women (P=0.022) (table 3).

**Table 3:** The relationship between mean of Gensini score and CAD RFs, drugs and other demographic characteristics in patients

**Characteristic**		**Mean of Gensini score**	**SD**	**P value**
Sex	Male	52.27	22.92	0.022
	Female	37.17	22.23	
Region of residence	Burgess	45.02	23.62	≤ 0.001
	Rural	5	-	
Educational state	Primary education	12.5	-	0.170
	Secondary education	39.82	21.75	
	Academic education	49.11	24.61	
Smoker (Yes/No)		48.33/43.97	20.38/27.72	0.583
Opium addiction (Yes/No)		43.00/45.10	12.72/24.05	0.903
HTN (Yes/No)		48.48/40.96	22.09/25.19	0.266
DM (Yes/No)		43.18/45.97	17.28/26.50	0.696
HLP (Yes/No)		49.92/40.12	21.56/24.99	0.144
Family History of CVD (Yes/No)		48.69/38.50	25.09/19.75	0.145
Drug history	ASA (Yes/No)	48.90/38.68	22.31/24.93	0.139
	Clopidogerel (Yes/No)	39.41/47.91	23.34/23.60	0.232
	Nitroglycerin (Yes/No)	46.71/44.15	24.39/23.55	0.721
	Statin (Yes/No)	48.26/42.26	22.83/24.37	0.376
	ACEI/ARB (Yes/No)	46.48/43.78	22.30/25.05	0.691
	Beta-blocker (Yes/No)	49.33/39.96	24.12/22.49	0.164
First presentation	MI (%)	96.34	10.23	0.084
	Unstable angina (%)	38.52	22.21	
	Stable angina (%)	49.72	23.87	
Vitamin D group	Sufficient	39.00	13.84	0.048
	Insufficient	26.00	12.72	
	Deficient	47.02	12.78	

**Fig. 1:**
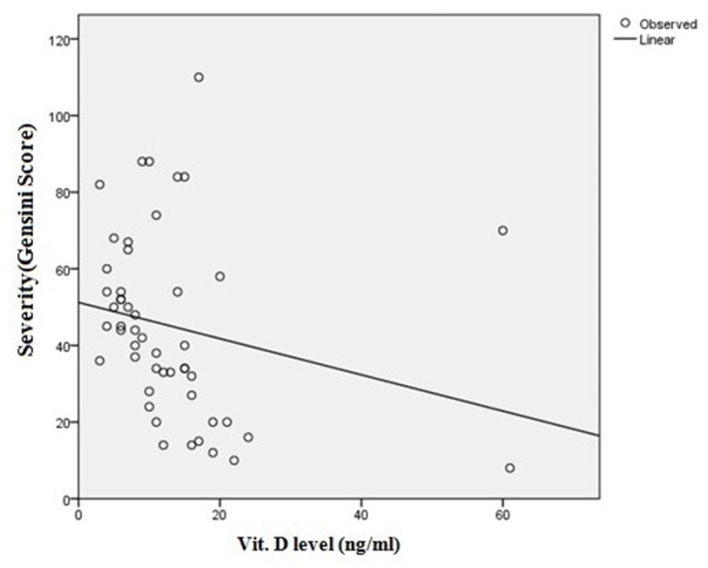
Correlation between vitamin D level and severity of CAD

The mean Gensini score of patients in the case group was 45.02±23.62. Regression analysis revealed a weak but significant inverse correlation between serum vit. levels and the Gensini score in these patients (P = 0.001 & R = -0.543) (fig. 1).

## Discussion

The most prominent finding of our study is that low serum vitamin D levels are associated with CAD. Furthermore, the more severe vitamin D deficiency/insufficiency is associated with more severe and extensive CAD. Our results also demonstrate that low serum vitamin D levels are also associated with some of the risk factors of CAD, including male gender, hypertension and positive family history for CVD. Another interesting finding of this investigation was that low serum vitamin D concentrations were associated with the use of aspirin. This may further confirm our main finding, since the use of aspirin could indicate a greater severity of the underlying CAD. The mechanism through which vitamin D can perform a protective role against CAD is not entirely clear. However, the proposed anti-atherosclerotic effect of vitamin D most likely is the most influential among them. Evidence suggests vitamin D is associated with decreased risk of endothelial dysfunction, calcification and stiffness in the arteries (13). Moreover, as Watson KE et al. demonstrated in their study, higher serum vitamin D levels are associated with a lower risk of coronary calcification (14). Studies have been controversial regarding the association between low serum vitamin D levels and increased risk of CAD. Many of them have supported such relationship. For instance, Akin et al. similarly used the Gensini score to evaluate the association between the extent of coronary artery stenosis and serum vitamin D status and reported that the Gensini score had an inverse association with serum vitamin D levels (r = -0.416, p<0.001) and therefore particularly stated that vitamin D is a predictor of the severity of CAD (15). Studies on Saudi and Indian populations have also produced similar results (16, 17). Some similar investigations have used the ‘synergy between percutaneous coronary intervention with TAXus and cardiac surgery’ (SYNTAX) score (18) instead of Gensini score to assess the extent of coronary involvement. An example of this is the study performed by Chen et al., where they also supported the association between the severity of CAD and low serum vitamin D levels (19). Similar to our study, theirs contained a relatively young population; however, a large portion of their study population had severe CAD, which makes their results almost impossible to generalize for all CAD patients. An interesting, recent study by Porto CM et al. has demonstrated a significant association between vitamin D deficiency and increased risk of heart failure in the elderly (20). This can also be linked to CAD, as CAD can also lead to, or exacerbate heart failure (21). Therefore, a normal serum vitamin D status can have a protective role against heart failure as well as CAD. Despite all this evidence, some studies have failed to demonstrate an association between low serum vitamin D levels and CAD. In a 2016 study, Dhibar et al. reported that although vitamin D deficiency had a high prevalence among patients with CAD, it was not associated with the severity of CAD (22). In addition, Mostafavi et al. performed an investigation on 224 patients undergoing coronary angiography and found no association between low serum 25OHD concentration on the extent of CAD.

 They even reported no association between hypovitaminosis D and the prevalence or risk factors of CAD (23). In another study, Degerud et al. also reported a negative association between serum vitamin D status and one-year progression of CAD (24). These results indicate that the debate continues. Larger and well-structured studies as well as meta-analyses seem necessary to reveal the true nature of any possible association between vitamin D status and CAD. 

**Study limitations: **While serum vitamin D levels vary with region, seasonality and altitude due to different levels of sunlight exposure, our data are from only one region and from patients of a single academic hospital. Therefore, the results should not be automatically generalized to other populations. Larger study populations from different geographical regions could result in stronger evidence. We recommend conducting prospective, randomized, controlled clinical trials evaluating the effects of vitamin D supplementation on the prevention and treatment of various cardiovascular diseases and prevention of relapsing CAD. 

In conclusion, Low 25-OH-D levels are associated with increased risk and extent of premature coronary artery involvement and its severity in patients who are 50 years old or younger. If this evidence is supported by larger studies in different geographical areas, early monitoring of serum vitamin D levels and vitamin D supplementation could be of great benefit especially for patients who are already at risk for premature CAD.
